# Digital Intrapreneurship: The Corporate Solution to a Rapid Digitalisation

**DOI:** 10.1007/978-3-030-53914-6_12

**Published:** 2020-06-25

**Authors:** Gifford Pinchot, Mariusz Soltanifar

**Affiliations:** 1 Hanze University of Applied Sciences, International Business School, Groningen, The Netherlands; 2 Loughborough University, School of Business and Economics, Loughborough, Leicestershire UK; 3 Nordhausen University of Applied Sciences, Chair of Digital Management, Nordhausen, Germany; 4 Seattle, USA; 5 Open University, Heerlen, The Netherlands; 6 Hanze University of Applied Sciences, Groningen, The Netherlands

## Abstract

For decades, intrapreneurship has been, and is still, promoted to employees as a way to capture the creativity and excitement of entrepreneurship, albeit with more resources and less risk. Intrapreneurship creates opportunities for individuals to be innovative and entrepreneurial within and for the organisation that employs them. The ways in which intrapreneurs act have not changed, unlike the business context surrounding them. Digitalisation has opened the path for new intrapreneurial opportunities; however, the amount of attention paid to the role of digital intrapreneurs within existing organisations is limited. We present our own definition of digital intrapreneurship and position our definition in the digital landscape where modern companies operate. This chapter outlines numerous ways to foster digital intrapreneurship, including a set of practical methods for managers to identify, and empower digital intrapreneurs. The chapter presents three case studies and discusses their practical implications for entrepreneurs and their teams.

## Introduction

PlayStation, iPod, Post-it^®^ Notes, and Gmail are all products of intrapreneurship. Introduced by Pinchot in 1978, intrapreneurship has long been promoted to employees as a way to capture the creativity, sense of purpose, and excitement of entrepreneurship, albeit with more available resources and less risk (Corbett 2018; Pinchot and Pellman 1999). Intrapreneurs are not merely talented speakers and polished PowerPoint presenters. They are individuals capable of making quick prototypes, testing ideas with potential customers, learning what works and what does not work, redesigning their products, testing them again, and pushing through or around whatever barriers are in their way. They are self-motivated, proactive, and action-oriented employees who take responsibility for turning an idea into a profitable business reality for their employer.

Digitalisation and digital transformation have opened new intrapreneurial possibilities. Digital tools and technologies are transforming business strategies and processes, firm capabilities, and key interfirm and customer relationships. These changes are not exclusively relevant to organisations focussing on digital products and services; they also affect how firms in traditional industries do business. Digital technologies are creating or changing most jobs and future growth opportunities. Digitalisation even transforms creative industries like music and film. Fundamentally, digitalisation puts enormous pressure on companies and individuals to reflect on their current strategies and explore new business and career opportunities (Rachinger et al. 2018). This is the ‘new normal’.

Intrapreneurs are as essential to corporate innovation as entrepreneurs are to start-ups, so most companies need many more intrapreneurs than they used to in the more stable times of the past. A firm’s capacity to foster intrapreneurial talent significantly affects its ability to address the many opportunities and disruptions caused by the digital transformation. For that reason, nowadays, an understanding of how a firm can create a corporate environment within which digital intrapreneurs can thrive is an essential leadership capacity.

According to recent studies, although digital transformation offers organisations numerous opportunities to involve intrapreneurs in seizing the opportunities made possible by digital technology, many of the platforms, designs, and tools that corporations use to encourage intrapreneurship are limited and ineffective (Reibenspiess et al. 2020). However, if managers can suitably locate digital intrapreneurs and accommodate their needs, organisations can function more effectively in a digitally transforming environment. This requires decision-makers to adopt entirely new ways of thinking, leading, and managing rather than simply approaching new processes with the same old mindset.

This chapter discusses the importance of digital intrapreneurs and explores the ways of identifying, surfacing, and empowering them within established organisations.

## The Relevance of Intrapreneurship to Digital Business 

This section defines intrapreneurship and digital intrapreneurship, describes intrapreneurial roles and behaviour, elaborates on the growth of digital transformation, and provides an overview of the subject.

### Defining Intrapreneurship

Definitions of intrapreneurship abound, each emphasising a different aspect of the term (e.g. Zahra et al. 2016). For example, intrapreneurship has been used to describe the following:

1. The entrepreneurial *initiatives* of a firm, viewing the firm as a whole as an individual actor
2. The *processes and structures* for managing intrapreneurs within an organisation
3. The *activities and behaviours* of intrapreneurs, their teams, and their sponsors.

In this chapter, to distinguish between these three aspects of intrapreneurship, we shall use the term *intrapreneurship* to refer to (a) the intrapreneurial activities of a firm as a whole and (b) the methods it uses to support and guide intrapreneurs. We use *intrapreneuring* to discuss the activities and behaviour of an intrapreneur and an intrapreneurial team as they work on developing and implementing innovative solutions. We will also use intrapreneurship as a general term to refer to all three abovementioned aspects.

Academic literature on intrapreneurship embraces innovative initiatives coming from employees when the initiatives come as responses to requests and challenges from a firm’s leadership and when innovations align with its strategy. Studies also recognise initiatives that began as bottom-up ideas and eventually received management approval. According to Pinchot (1985):

‘[Intrapreneurs are] any of the ‘dreamers that do’. Those who take hands-on responsibility for creating an innovation of any kind within an organization. The intrapreneur may or may not be the creator or inventor but is always the dreamer who figures out how to turn an idea into a profitable reality’ (p. ix).

Pinchot later defines one particular kind of intrapreneurs (1987): the ‘in-house entrepreneurs, those dreamers who can increase the speed and cost-effectiveness of technology transfer from R&D to the marketplace’ (p. 14).

Our definition of intrapreneurship is somewhat broader than general usage. Writing about intrapreneurs often focusses on the people within an existing organisation who develop innovative products or services provided to external customers. However, people can use their intrapreneurial spirit for many things other than new externally focussed products and services, instead concentrating on developing better ways to make, improve, and sell products and services. Although Pinchot’s perspective includes both the intrapreneurial actors (i.e. intrapreneurial leaders and teams and their sponsors) and the ways corporations could encourage intrapreneuring, most studies on intrapreneurship and the often interchangeably used term ‘corporate entrepreneurship’ have focussed on organisations and not individuals (Soltanifar 2016). Moreover, throughout the past decade, studies on intrapreneurship or corporate entrepreneurship have been dominated by analyses of firm-level contributions, that is, the instances where firms acted as entrepreneurs (e.g. Lumpkin et al. 2009; Rauch et al. 2009), with only a few exploring the individual-level or team-level perspectives.^1^ Until now, no studies had expressly modelled the individuals’ intrapreneurial behaviour within the context of digital intrapreneurship.

### Intrapreneurial Roles and Behaviour in Organisations

Pinchot and Pellman (1999) recognise five distinct roles that are essential for managing innovation: (1) an idea generator, or an inventor, (2) an intrapreneur, (3) an intrapreneurial team member, (4) a sponsor, and (5) an innovation climate maker. Although all five roles need to coexist to result in successful innovation, the permitted space, unfortunately, does not allow us to discuss all of them; thus, in this chapter, we focus solely on the roles of the intrapreneur and the sponsor and their contributions to digital intrapreneurship.

Intrapreneurial activities range from large interventions, such as creating new business ventures and changing the strategic direction of a company, to smaller changes, such as developing new products, services, and technologies and improving existing products and processes. Intrapreneurs, like entrepreneurs, prefer to act without having to prove that their attempts will necessarily be a success (Pinchot and Pellman 1999). Instead, they want to find out what will work through a series of experiments, learning scenarios, and redesigns. They are prepared to encounter obstacles and setbacks, learn from them, and adjust their initial assumptions according to any new information. Intrapreneurs operate across the boundaries of organisational units, which is often necessary, since many new ideas require changes in more than one aspect (Pinchot 1985).

Intrapreneurs’ anticipatory behaviour aimed at creating, and later implementing, new ideas for their organisation increases its capacity to respond to new opportunities and external developments (e.g. Gawke et al. 2017). According to Deloitte (2015), this action-oriented intrapreneurial behaviour is often combined with a strong business focus and a relationship-building skill set, enabling intrapreneurs to actively sell their ideas within their corporations and thus drive their implementation. Without such skills, intrapreneurs might lack internal sponsorship and, regardless of their creative spirit and vision, fail to convince management to let them proceed. Intrapreneurs operate within their respective companies and are thus acutely aware that they will never act as independently as entrepreneurs (Deloitte 2015).

Like the role of intrapreneur, the role of the sponsor has been extensively discussed in the literature on innovation and corporate entrepreneurship. Sponsors serve to ensure that the intrapreneurial projects they finance are legitimate and supported (e.g. Hayton and Kelley 2006). They help intrapreneurs to gain access to any resources they need for their ventures (e.g. Day 1994). Good sponsors are able to distinguish the real intrapreneurs from the ‘promoters’ who look and sound good but fail to get the job done. Once they select an intrapreneur to support and trust, sponsors protect and coach them on future strategies (Garud and Van de Ven 1992).

This demands a lot of the sponsors’ time for each intrapreneur, so if many innovations are needed, as they are in today’s disruptive environment, many sponsors are needed to coach and protect the many intrapreneurs that drive those innovations. For this reason, it is important that executives delegate discretionary time and budget to lower-level managers so they can support the many needed intrapreneurs (Hayton and Kelley 2006).

### The Growth of Digital Transformation and Its Implications for Intrapreneurship

Many emerging digital technologies are called exponential because every few years their capabilities are doubled. Because they are rapidly becoming impactful, exponential technologies like the Internet of things (IoT), artificial intelligence (AI), machine learning (ML), 3D printing, robotics, and blockchain are creating many new opportunities in most industries almost every year.

IoT, for instance, opens up new possibilities for product development, logistics, and improved business processes (Phaneuf 2020). IoT also provides powerful tools for tracking the quality, the ownership history, and the social and environmental attributes of the supply chain. This might greatly increase the capacity of organisations to manage their supply chains and address the sustainable development goals set by the United Nations.

AI enables users to process huge amounts of consumer data accumulated from various customer interactions to provide new and enhanced customer-centric insights, which are useful for idea generation, advertising, surveillance, and the invention process (Newman 2019). Machine Learning, a type of AI, lowers the costs of prediction and problem diagnosis, which are inherent to all business decisions (Forbes Technology Council 2019). Blockchain provides access to various markets, smart contracts, finance innovation opportunities, and enhanced security and competitiveness strategies (OECD n.d.). However, these powerful exponential technologies, despite their numerous benefits, may cause undesirable results. First, such exponential technologies might radically reduce consumer privacy and potentially induce totalitarian control through enhanced surveillance mechanisms. Second, they may also increase the criminals’ ability to conceal illegal activity and transfer the right to create money away from governments to private entities, which might significantly impact the distribution of wealth. Both of these possibilities come with ethical and political issues that businesses will have to manage.

Although digital transformation is currently impacting a large variety of businesses, we have noticed a limited display of attention towards the role of digital intrapreneurship within traditional industries. Nevertheless, digital intrapreneurship plays a significant role in such industries by increasing production speeds, streamlining logistics, managing processes, lowering costs, handling supply chains, supporting low-cost customisation, managing risks, and allowing companies to build more responsive relationships with customers.

To seize these opportunities, even the most traditional manufacturing businesses must initiate systems and foster corporate cultures conducive to digital innovation. This is not just about coding or system design skills; rather, this transition requires an understanding of how digital natives live. Most digital natives are millennials or younger people, who are born after 1982. This is not to say that older people cannot drive digital innovation—many can; however, the volume of talent required to deal with the speed of contemporary digital transformation means that even mid-sized companies must recruit, motivate, and retain many young digital intrapreneurs. Digital natives understand the ways in which emerging technology can be, and is, used (Rossi 2019).

What are digital natives looking for? Deloitte (2019) has recently conducted another round of their ‘Millennials Survey’ and suggested that millennials have the following expectations:

1. Work that is aligned with their sense of purpose
2. A chance to make a significant contribution before they are 50
3. Freedom to choose what projects to work on
4. Freedom to act and make decisions about their work without frustrating delays caused by waiting for permission
5. Work that aligns with a desire to make the world better, as well as producing profit.

These demands do not fit well with command-and-control management approaches or shareholder-value-only objectives. However, these demands do not come from an unreasonable sense of entitlement by the young. They are what employees need to get the digital innovations and the other increasingly creative, intrinsically motivated and self-guided work of the twenty-first century done.

Older managers, not realising that the nature of work is changing, might think that the demands of the young are absurd; however, most talented digital natives will stay in an unsupportive company for only as long as it takes to establish a good résumé entry and then leave to work for another employer who will be more willing to accommodate their needs. Many older managers find it frustrating to manage these young people, who do not seem to behave ‘the way the employees ought to’. And yet, these young people and many of their behaviours are essential for the development of a robust strategy of digital transformation.

### Putting It All Together: Digital Intrapreneurship

The broadened definition of intrapreneurship presented under 2.1 is particularly pertinent to a discussion of digital intrapreneurship. Digital intrapreneurship is any intrapreneurship that uses digital means as a critical component of its innovation initiative. The innovation itself can be a new digital product like Google’s email client or Amazon’s cloud storage; however, it can also be exemplified by the use of digital technology to do what the company already does, but better, cheaper, and faster.

The latter kind of innovation is the most important form of digital innovation for companies in traditional industries. For such companies, digital innovation is not about new digital products or services but rather about the better ways to market, relate to customers, create operational efficiencies, and use exponential technologies such as 3D printing or genomics to perform the current processes much better, faster, and cheaper. For example, digital intrapreneurship includes using AI to optimise scheduling in a trucking firm or image interpretation in health care. It can also be used to market non-digital products, such as Amazon’s online sales of physical products, or design a new physical product, such as a new medication or an airplane. Much of the innovation of Boeing 777 was done using digital tools, which allowed to rapidly design a better integrated airplane, thereby streamlining production.

Continual improvement of operational processes is still best done using the total quality method and its descendants like Six Sigma; however, breakthrough process improvements are mostly done by digital intrapreneurs. If one looks closely at continual improvement processes, one will often find that they create an environment where employees express a higher degree of initiative that resembles an intrapreneurial spirit.

Google’s use of ML to improve their language translation services is good example of a radical product improvement made possible by digital intrapreneurship. Google was already delivering machine translation to customers; however, a small team of intrapreneurs overrode the traditional methods of its translation engines with a statistical ML approach. The outcome of this decision was an exponential improvement in the quality of translation, which was so striking that it caused Google to promptly stop working on improvements reliant on older methods (Lewis-Kraus 2016).

Quite often, digital intrapreneurship offers innovation opportunities that can create major transformations in terms of efficiency or customer relationship with a very modest investment. This creates a large number of high return-on-investment (ROI) intrapreneurial opportunities by developing personalised customer relationships, collaborating with suppliers, taking more data-driven decisions, automating diagnostics, managing natural resources like energy or water, and optimising logistics and process control.

*Digital intrapreneurs* are employees who use their entrepreneurial spirit for the benefit of their employer and simultaneously to give meaning to their work by implementing their ideas to produce impactful digital innovations.

Digital intrapreneurs must possess the skills to identify new digital-technology-enabled business opportunities and bring them to fruition, either as a new concept altogether or as an existing, but transformed, business system (World Bank Group 2016). Even though many companies seemingly focus on using innovation to drive commercial growth, what many of them miss is a corporate culture of innovation and a safe and supportive environment for their digital intrapreneurs. Creating that environment requires supportive managers to protect and coach one or several intrapreneurs that they personally trust and want to empower. Many such managers produce many empowered intrapreneurs. The collaboration between intrapreneurs and sponsors can be facilitated by a culture that permits them to act. Together these factors can lead to great levels of digital innovation.

## Digital Intrapreneurship Model

Based on our review of the relevant literature, as well as our practical experience, we offer the following conceptual model that enables established organisations to surface, identify, and empower digital intrapreneurs to drive digital innovation. Finding, retaining, and supporting digital intrapreneurs, including millennials and GenX digital natives, is a core competency in our times.

Every organisation has control systems that create barriers that slow down intrapreneurs or stop them entirely. How, then, does innovation take place? In every organisation we studied, the key to innovation has taken the form of courageous managers who guide, protect, and clear the way and get resources for one or several intrapreneurs with whom they have close and trusting relationships. In effect, to those who put up barriers that block intrapreneurs, they say, ‘I have checked this team out, and they are on the right track. They are acting responsibly. Let them pass’. We call these courageous managers ‘sponsors’.

**Fig. 1:**
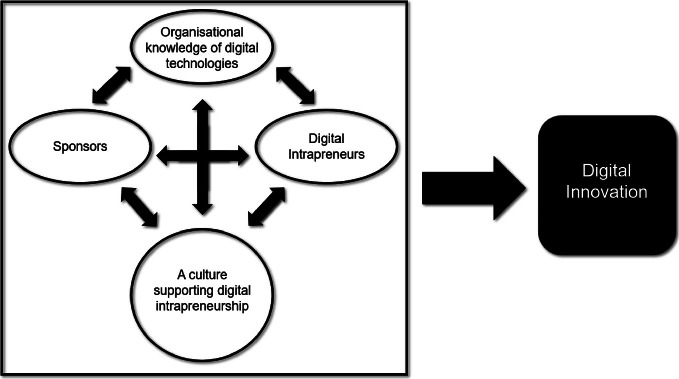
Digital intrapreneurship model—the corporate solution to a rapid digitalisation

Nowadays, organisations cannot flourish without an organisational knowledge of digital technologies. Some of this knowledge is provided by digitally competent employees. The large issue of how an organisation can learn to act using the knowledge of digital technologies is unpacked below (Fig. 1).

Next, we shall elaborate on each component of the model.

### Sponsors: The Key Factor for Supporting Digital Intrapreneurs

The first factor positively affecting digital innovation is a sufficient number of good sponsors. Often, when business leaders call for more digital innovation, it does not happen. When it works, how does the intent of the leaders to support digital intrapreneurship go through the ‘clay layer’ of middle managers who are usually driven so hard to achieve short-term goals in established systems that they have no time for new ideas?

In practice, we have found that the answer lies in a special class of managers who, because of their own intrinsic motivation and their relationship with intrapreneurs and their teams, choose to go out of their way to help the intrapreneurs. They spend their political capital to support the intrapreneurs even though it is not their ‘day job’. As mentioned above, these altruistic managers are called ‘sponsors’. Sometimes, they are called champions, but that term is a bit ambiguous because it is often applied not only to the sponsors, who champion the intrapreneurs, but also to the intrapreneurs themselves, who champion their ideas. Thus, the term ‘sponsor’ is clearer.

Sponsors spend time with intrapreneurs and coach them on both the commercial and the political issues and strategies. They stand up for the intrapreneurs when they are not present and help them access any necessary resources. If an innovative solution works in a given company, it is almost always due to a close and trusting relationship between a self-motivated team of intrapreneurs and their management sponsors. That combination is what moves innovation forward through the inevitable resistance of any corporate system.

Organisations can facilitate sponsorship in several ways. First, companies can train managers to be effective sponsors. This training includes both a description of what a sponsor must look for in an intrapreneur and some dos and don’ts for managing them.

Second, organisations can promote sponsorship by authorising lower-level managers to serve as effective mentors. Companies can provide managers with discretionary budgets to fund the early stages of innovation. These budgets do not have to be large to have a positive effect. Often a rapid prototype and a little travel money can be enough for testing an idea and gathering enough data to make a strong case for pursuing it further.

Third, companies can hold managers accountable for sponsoring innovation. They can feature sponsoring intrapreneurs as a responsibility on the list of the managers’ key performance indicators (KPIs). Human resources can assess the sponsors’ performance are by asking successful intrapreneurs: ‘In your darkest hour, among your management, who supported you and helped you deal with whatever barriers were in your way?’ The individuals mentioned in the answer to that question are the true sponsors. Then, counter-intuitively, when the good sponsors are identified, they should not be celebrated.

Great sponsors give credit to everyone around them, so celebrating them publicly will annoy all the others who ended up getting credit for what the sponsor did. This will create jealousy and limit the sponsor’s future effectiveness. It is important to value what good sponsors do, but this can be done by congratulating them privately and, like succession planning, by keeping a secret list of good sponsors and promoting them whenever possible. As they rise, the true sponsors can be even more effective in supporting intrapreneurs and the culture that makes them effective.

Fourth, companies can measure their innovation outputs. At 3M, division leaders were held accountable for the number and quality of the innovations coming out of their division. The innovations were graded by the company’s innovation rating team from minor improvements to those innovations that could create disruptive products for years to come. The leaders were not prompted to be innovative themselves. To get a good innovation score, a division needed to foster a corporate culture where intrapreneurs could thrive; this was measured using the innovation output of each division. This made having high-quality intrapreneurs and sponsors in their divisions valuable to general managers, who therefore created conditions conducive to intrapreneuring.

The best sponsors are motivated intrinsically, rather than extrinsically. They support intrapreneurs because they buy into the intrapreneurs’ ideas and passions. Helping the intrapreneurs gives them meaning and provides them with valued professional relationships. The most effective sponsors are not driven by their ego. In extreme cases, they might have already reached the highest level they could expect to achieve in their career, so now they are giving back to younger innovators who remind them of their previous selves.

Many processes aimed at innovation fail. Idea contests bring out lots of ideas but rarely lead to successful implementation. The result is that they give hope to numerous employees only to crush them eventually. Rather than increasing employee engagement, they cause a short-lived increase in it followed by a long-term decline.

Formal processes like Stage-Gate, which at least intends to provide a pathway to commercialisation, should work better. However, much too often, due to delays between review cycles and an excessive focus on secondary information rather than on the testing of quick prototypes and intrapreneur assessment, they tend to slow down and halt innovation instead of accelerating it. They often become a process that creates more ways to say no and kill an idea rather than building a system for supporting intrapreneurs.

Significant decisions are made by committees, but no one has the ability to dig deep enough to understand the most difficult ideas. Committees may eliminate bad ideas, but they also tend to reject highly innovative and disruptive suggestions because they are hard to understand. What tends to survive are mediocre copycat ideas.

What works for selecting and supporting innovations is not a process, but rather a large set of close relationships between intrapreneurs and their sponsors who have some clout and influence and who trust, spend time with, and give extraordinary support to specific intrapreneurs. These sponsors get to know their intrapreneurs, their team, and their ideas very well. They are in a good position to evaluate the intrapreneurs and their proposed innovations. Their judgement on what the company should invest in is better than that of the committee; additionally, they can help the intrapreneurs to improve their ideas using well-informed questions and coaching.

Successful intrapreneurs, digital or not, often have several committed sponsors occupying different positions at different levels of the organisation. Creating and managing a coalition of sponsors is thus a core intrapreneuring skill.

Consider these facts together:

1. Each intrapreneur usually requires several collaborating sponsors to support and protect their interests and ideas.
2. Many intrapreneurs are needed to deal with the threats and opportunities rapidly generated by exponential digital technologies.
3. Sponsoring is an intimate relationship, which makes it time-consuming; that means that each sponsor can only protect one or few intrapreneurs.

These facts imply that each company needs a great number of sponsors to support the many intrapreneurs necessary to face the era of rapid digital innovation.

Senior leaders cannot possibly provide the volume of sponsorship sufficient to address the opportunities and threats created by exponential technology. Their role is to create the systems and a culture that empower middle managers and even first-line supervisors to serve as effective sponsors. Any intrapreneurship programmes that necessitate the blessing of senior leadership for individual innovations will fail for simple numerical reasons. In the age of digital innovation, authority to give the green light to innovation must be delegated.

### Organisational Knowledge of Digital Technologies

The second factor driving digital innovation is the organisational knowledge of digital technologies. What does this mean? It means that an organisation, as an entity, makes decisions and takes actions as if it understands and is gracefully creative with its use of digital technology. It is not just about how many people with digital skills are employed by a given company; rather, what matters is how an organisation responds to digital opportunities and threats. That is what matters. There are two main elements of the organisational knowledge of digital technology:

1. The presence of a sufficient number of members of an organisation who understand digital technology and can create and implement all the innovations necessary to drive the digital transformations needed by that organisation
2. The way an organisation makes decisions and takes necessary actions to exhibit a fluid knowledge of digital technology.

Some organisations succeed in both aspects. Smart digital thinking pervades every aspect of their corporate functioning. As for the rest of modern organisations, it is unlikely that their largest barrier to digital innovation is a lack of people with the knowledge of digital technologies.

Companies today hire many digital natives. However, if digital innovation does not take place, the issue is more likely that a company is blocking the intrapreneurial spirit of its digitally competent employees, while those with significant digital talent are leaving the organisation or are disengaged and demotivated. See Case 2 below for more on this situation.

The major problem preventing most organisations from acting from a place of understanding of digital technology lies in their management. If management has neither the sufficient understanding of nor the familiarity with digital technology, how can it rapidly foster the organisational knowledge of digital technology?

1. Certainly, widespread education about digital technology is one part of the answer; however, it will probably take time to help the senior management reach the required level of understanding of and familiarity with digital technology where it would be able to properly assess any proposed digital innovations. There are faster approaches to the issue in question.
2. If more senior-level managers put their trust in selecting mid-level managers as sponsors, the growth of digital organisational competence within a company would significantly increase without the need for substituting any senior managers. A cultural transformation aimed towards increasing professional trust is necessary to empower intrapreneurs, and it can also increase the company’s organisational intelligence in digital matters much faster than if the company tried to foster a profound digital competence among its senior executives. However, the senior members are still needed to provide wise advice about the core of the business that takes years to develop.
3. If a company learns to give more weight to the character, competence, and track record of its intrapreneurs, with slightly less focus on the initial quality of their ideas, then their sponsors will be able to make better decisions even without a detailed knowledge of digital technologies. In this scenario, the sponsors may augment their understanding of technology by knowing how to recognise and relate to true intrapreneurs.
4. Senior executives and middle managers can build a small set of digitally competent advisors to help them understand and assess proposals related to digital transformation. These advisors may not be highly ranked and may function best in a formal role as coaches on digital technology, with any advice they offer on more strategic matters being done informally so as not to disrupt the sensibilities of the chain of command.
5. As demonstrated in Case 2 below, a company can acquire digital talent through acquisition; however, if it does not learn how to create an appropriate working environment for its intrapreneurs and the emerging creative work of the twenty-first century, they will soon be gone. A company must not impose its culture on any acquired entities; instead, it must learn from the acquired businesses about how they can better manage their own operations in this new digitally transforming world.
6. If a company has already developed a good organisational capacity to understand what digital transformations it should take on, then it can hire or partner with external organisations to complete the most technical aspects of its digital innovation plans. This requires a culture that knows how to deal with external entrepreneurs and intrapreneurs in vendor organisations.

Big companies in this era often need to partner with smaller digital innovators. To utilise the potential arising from the know-how of external digital entrepreneurs, big firms need to operate and make decisions at or close to the speed of their entrepreneurial partners. Otherwise, the entrepreneurs—or even the digital intrapreneurs within the larger partners—might become frustrated with partnering with a slow-moving firm or, worse even, take advantage of it.

The only way to achieve the necessary operating speed is to delegate the responsibility for managing relationships with external partners to a team of intrapreneurs, who are driving the part of the innovation being done by the larger firm, and let them make decisions with their external partners without constantly waiting to get their permission to make the next move.

### Managing Digital Intrapreneurs: A Core Competency for Digital Innovation

The third method for supporting digital innovation lies in high-quality management of digital intrapreneurs. Motivating intrapreneurs is not necessary; instead, it is sufficient to merely not demotivate them by preventing them from taking their ideas further. A business leader can and should ask complex open-ended questions with the goal of helping them avoid trouble, but, whenever possible, trust them to come up with the right answer by themselves. A leader should also let them make non-fatal mistakes if these questions do not help them see the faultiness of their plan. After all, their plan might be smarter than expected.

A business leader should also be a good friend, who is concerned about the well-being of their innovations. By asking questions about the possible weaknesses in their plans while still letting them come up with the answers, a leader can support their intrinsic motivation. If he or she tells them what to do, their motivation will shift towards getting the permission of the manager (an external motivation), and the spirit of intrapreneurship will be lost. One must let them be the driving force, support them, and clear the path in front of them.

Intrapreneurs require an unusual level of freedom to be effective. This means managers have to trust them to rapidly make decisions about the development of their innovations without having to wait for permission or review. But one cannot trust everyone equally; thus, determining which digital intrapreneurs are worthy of trust and thus which ones to fund is critical to cost-effective digital intrapreneurship.

According to Pinchot (1987), ‘Venture Capitalists say, “I’d rather have a class A entrepreneur with a class B idea than a class A idea with a class B entrepreneur”’ (Pinchot 1985, pp. 15–16). The same logic applies to choosing the right intrapreneurs and innovations to invest in.

Pinchot (1987) continues:

‘Picking the people with a passion, attitudes and talent for making the idea work is more important than picking the right plan. … Corporations can greatly increase their return on innovation efforts by moving the emphasis in their innovation management efforts from selecting the right plan to selecting the right team to trust’ (Pinchot 1987, p. 14).

The reason why people are more important than ideas is because almost no innovative idea will work in its original state. No one is that smart and foresighted. Investors need to have an appropriate team that can learn from its setbacks, experiments, and surprises and use that information to develop a functioning plan. For this reason, when deciding which digital innovation to invest in, the intrapreneur and their team serve as two most important factors.

A core part of that task involves seeing the difference between the real intrapreneurs and the individuals that venture capitalists call ‘promoters’. Promoters are posers who talk a lot but lack the grit, persistence, courage, and intrinsic motivation to push through all the barriers, setbacks, and changes that will inevitably arise when they will try implementing an innovative idea. Promoters are driven by their ego and a desire for status rather than a genuine commitment to a transformational idea. When things go wrong, they will try to gloss over the problems instead of digging deeper to nip them in the bud. They will try to embellish their ideas instead of acknowledging the need for change. They will redirect supervisory attention to how great it will all be at the end of the journey instead of trying to dig the problem out.

Real intrapreneurs, conversely, are very interested in the pathway leading to the implementation of their ideas. If a supervisor suggests anything that might get in the way of implementing their ideas, they will take it seriously. They will, if necessary, ask questions to understand any related concerns. Alternatively, since they have probably already thought about the potential problems, they will be happy to tell about the ways of mitigating or circumventing the obstacle in question. Moreover, they will be interested in their supervisor’s thoughts about the issue.

Following are some things to look for when deciding whether to back a proposal for a digital innovation (Table 1).

**Table 1:** Ten criteria for approving an intrapreneur’s proposal for digital innovation

Criteria	Characteristics
Collaboration	Digital innovations usually span many functions and business units. Thus, collaboration with other business functions, even if they initially occupy hostile silos, is a core intrapreneurial requirement *Does the intrapreneur collaborate effectively?*
Deep involvement with the steps leading implementation	Real intrapreneurs are capable of envisioning the pathway to success as well as the final outcome of the innovation *Is the intrapreneur thinking clearly and in detail about how to implement their idea?*
Honesty	Honesty is a core character trait of successful intrapreneurs. Intrapreneurs may bend the rules or even break them to get something done, but they will always be open and honest with their potential sponsor. Moreover, they will not lie to others, even though at times they may hold their cards close to their chest. If they are not honest and open with their sponsors, they are probably not honest with themselves, which means that they will ignore data that does not support their desired expectations. The result of such an approach will almost certainly be a failure *Are they honest with you?*
Long- and short-term goal setting	Intrapreneurs set goals and assess their progress against them. If they are missing their targets, they want to know why. This helps them stay focussed, experimental, and realistic *Are they interested in assessing their own performance?*
Moderate risk-taking	Successful intrapreneurs take on challenging initiatives but do everything in their power (e.g. early tests of rapid prototypes) to reduce the accompanying risks. They are not gamblers; however, security is not their prime motivator either. The intrapreneurs’ sense of security comes believing that they and their team have the ability to handle whatever problems that might arise *Are they good at managing risks?*
Motivation	Real intrapreneurs are intrinsically motivated—motivated from the inside by their values, vision, and purpose. Even though, like anyone else, they like to be paid well, money is not the reason for their new ideas. They innovate because they think their innovation matters above and beyond money. Money is a way of keeping score on how well they are doing in pursuing their vision, but it is not the reason for pursuing the innovation in the first place. This does not mean that they do not need to be paid well. They (particularly the talented intrapreneurs born after 1980) will leave if they find themselves paid substantially less than their peers who are just climbing up the conventional managerial ladder at a leisurely pace *Is their motivation deeper than money or promotions?*
Optimistic, inspirational leadership	Intrapreneurs do not have the resources to materialise their vision. Hence, they must inspire others to volunteer and help them construct their dream. Eventually, when they face a big setback (which is almost inevitable in every innovation), they may not claim that they know the solution but rather express their genuine belief that their team will find a way around the issue. If they cannot maintain that optimism, they will lose their followers *Do they attract proactive and inspired followers?*
Persistence	A predominant characteristic of both intrapreneurs and entrepreneurs is a deep persistence. If a senior executive puts a stop to their idea, a promoter will simply switch to another idea to get back in the executives’ good graces. Real intrapreneurs are not interested in pleasing executives, so they do not give up. Instead, they find support elsewhere or build a plan to change the executives’ mind *Treat persistence as a positive indicator*
Team building	Intrapreneurship, particularly digital intrapreneurship, is not a solo sport. Most innovations require a team. For instance, most digital innovations require a team that contains at least a system architect and a coding manager. Most teams also need a sales and marketing person, and that is just the beginning *Can the intrapreneur attract a team and run it effectively?*
Technical capabilities	A digital intrapreneur does not have to be a star technical talent. In fact, many intrapreneurs will have balanced skills and will usually be comfortable with new technologies and capable of understanding and working with those who excel in detailed tasks *Does the team have the necessary technical skill set?*

Of course, the idea that the intrapreneur wants to pursue is also part of the evaluation process; however, a good idea without a good intrapreneurial team to implement it is of very little value. Too often, in corporate decision-making, the quality of an intrapreneurial team and their commitment to an idea take a back seat to the analysis of the idea. Even worse, sometimes, passionate intrapreneurs are replaced with bureaucrats who lack both the passion for the idea and the intrapreneurial mindset necessary for innovative success.

### A Culture Supporting Digital Intrapreneurship

The fourth factor contributing to effective digital innovation, in addition to intrapreneurs, sponsors, and organisational competence in digital technologies, is a supportive culture. Creating and nurturing a culture where digital intrapreneurs can thrive in the organisation is a core capability for facing the world of exponential digital innovation. Building such culture is not about creating intrapreneurs, since they already exist, often concealed, within established organisations. It is, however, about discovering them, showing them that manifesting intrapreneurial behaviour is safe, and supporting and empowering them. Instead of engaging in an academic discussion of corporate culture, let us display a number of practical activities that can be undertaken to facilitate a digital intrapreneurial culture within an established organisation and give hints on how one can succeed using those activities: applies to the entire chart (see Table 2).

**Table 2:** Activities within established organisations supporting digital intrapreneurs

Activity	Implementation suggestions
A vision of the organisation’s overall destination and goals	Create and communicate a vision that inspires digital intrapreneurs, let them know about any challenges faced by the company, and invite them to come up with digital solutions to those challenges. (Also, keep the door opened for divergent ideas with small budgets, as these may find their application in future.)
Active involvement of management and senior leaders	Keep talking about digital intrapreneurship. Watch for and celebrate successes. Reward managers when their people innovate, so that they do not steal their subordinates’ ideas. The H in Help looks like II in the pdf. Help intrapreneurs develop the leadership skills they need. Build a culture that supports intrapreneurship
Support of digital intrapreneurs	Intrapreneurs are as essential to corporate innovation as entrepreneurs are to start-ups. Cherish your intrapreneurs. Build a culture that supports them. Build an intrapreneurial career path. Support implementation by digital intrapreneurs, not just idea inventors and early development specialists. Allow employees time to think and test their ideas
Support of cross-functional teams	Digital innovations often involve changes in the way things are done in the non-digital parts of the organisation. Support cross-functional teams by assigning team members from non-digital sectors of the firm. Give the teams time and the ability to make decisions together. Support the team’s decisions instead of letting the decisions propel into turf battles between the functional seniors
Creating a sponsorship culture	Management sponsors who select, coach, protect, and allocate resources to intrapreneurs are the primary support for intrapreneurship. Innovation is more about this relationship than any other process Train and expect managers to sponsor one or more intrapreneurs whose character and innovations they trust Make sponsoring innovations a central part of the corporate culture and every manager’s job. Include sponsoring success into management KPIs
Widespread intrapreneurial training	Deliver a short course for everyone to know what intrapreneurs are, what they do, how they act, and the ways they can be effective. Let managers know about the support that intrapreneurs require and how the managers can provide it. Managers, executives, and individual contributors can all attend the same two-hour online training lesson, so they all get to see how the intrapreneurial system works
Idea exposition	Organise both the online and the in-person idea expositions that help intrapreneurs to share their ideas and attract others to join their intrapreneurial teams. Management can also tour the expositions and look for intrapreneurs to support. Expositions can result in the creation of teams that attend innovation accelerators
Use of digital innovation accelerators	Accelerators are action-learning workshops that help teams of intrapreneurs develop their ideas, increase the quality of their teamwork, and bring out their intrapreneurial spirit. They can be full time or part time; however, part time is more common in the corporate world. The workshops usually range from six weeks to six months or longer
Delegation of discretionary time and resources to the lower levels	Today, computers can monitor every minute of an employee’s time and document their use of resources. This hinders the casual experimentation, daydreaming, and ‘fooling around’ that often serve as the source of innovative breakthroughs Allocate discretionary budget and time to the lower levels: to individual contributors, their supervisors, and lower-middle management. Offer employees the option of spending some of their time and modest supplies on side projects of their own choice. Let supervisors and lower-level managers sponsor the early stages of innovation from their own discretionary budgets
‘Sandbox’ or ‘seed’ fund allocation	Seed funds are pools of discretionary finances reserved for early-stage innovations. Create small local seed funds distributed throughout the company. Seed funds create a route circumventing the bosses who block employees’ early-stage ideas. It is not just a monetary grant; it is an implied permission to work on an idea Let any employee apply and, if they succeed, give them some time off to pursue the idea. Seed funds generally only award small grants for a rapid prototype test or similar purposes
Boundary crossing	Since digital innovation tends to cross boundaries, digital intrapreneurs need permission to cross these boundaries and be encouraged when they ask for help. This cultural attribute of generosity can serve as a powerful booster of digital intrapreneurship. Reward cross-boundary generousity. Ask intrapreneurs, ‘Who helped you in the early days?’
Anticipation and failure acceptance	Do not punish intrapreneurs for any original mistakes committed in pursuit of an innovation. The best pathway to success involves making your mistakes faster and cheaper and then quickly learning and adapting. Have meaningful ‘good try’ recognitions and rewards. Make sure these rewards are viewed to be positive by the recipients. Venture capitalists like to invest in entrepreneurs who have experienced both failure and success
Articulation of a digital culture	Establish your company’s digital vision. Talk about the kinds of things you hope that digital innovation can do for the company. Say that you need help to make those things happen
Small beginnings	Corporate strategists often discount the value of small innovations, sometimes saying that they are of no significance. However, small beginnings often pave the way for the arrival of major opportunities and serve as places from which to explore and learn about a new possibility. The lean start-up model prescribes a rapid testing of ‘minimal viable products’. Value small beginnings and intrapreneurial investigations of new possibilities using small budgets. Then, if particular ideas start to work, spend more on scaling up what already shows signs of success
Assessing the innovation output	Assess the innovation output of each business unit. Create a scale of how impactful an innovation is with many points allocated for disruptive innovations. Give units overall innovation scores, and hold them accountable accordingly Let multiple units get credit for the same innovation if they all made substantial contributions. This promotes cross-organisational cooperation
Developing new ways of organising work	Keep a backbone of hierarchical control, but release innovative structures from it. Create a convenient platform for a network of self-organising and self-directing intrapreneurial teams that function in the chain of command. Let empowered intrapreneurs select their team members from those who wish to join. Let the teams stay together and take on projects cooperatively. When possible, let intrapreneurs be responsible for the execution of their own initiatives
Giving rewards	Build an intrapreneurial career path that provides successful intrapreneurs with good salaries, sufficient time, and budget to innovate again. Freedom to work on their next ideas is the most effective reward for intrapreneurial success. Reward the whole team, not just the leader. Do not rank people within innovation teams; an ‘all boats rise and fall together’ reward system promotes teamwork

## Examples from Practice/Case Studies

The case studies below describe the practical ways of increasing digital intrapreneurship.

### Case study 1: Finding, surfacing, and empowering digital intrapreneurs at Deutsche Bahn

The Deutsche Bahn (DB) Group is one of the world’s leading mobility and logistics companies. DB employs some 331,600 people around the globe, including roughly 205,000 in Germany (Deutsche Bahn 2019). The company trusts in the innovative potential of its employees and believes in unleashing their potential to develop corporate start-ups. Its programme motivates the employees to work on solutions for problems that they have identified. The programme enables teams of employees and external team members to test and develop their ideas, potentially creating an internal business unit or even and external company. Within the structured programme, desirability, feasibility, and viability are considered to be the focal points.

The corporate entrepreneurship department with its intrapreneurship programme, ‘DB Intrapreneurs’, is part of the Chief Digital Officer unit of the DB Group. Launched in March 2017, DB Intrapreneurs is a fundamental part of DB’s digital and cultural transformation strategy across all its divisions. As internal incubator, the purpose of the programme is to offer all employees the possibility to develop their own digital business models and products in an empowering environment. Moreover, participants gain entrepreneurial mindset and skills.

DB designs and operates the transportation networks of the future. Through the integrated operation of the traffic and railway infrastructures as well as the economically and ecologically beneficial connection of all modes of transport, the company focusses on the transportation of both people and goods. In 2017, it held a market share of 67%. DB’s target is to increase punctuality, quality, and reliability of its transport. Its efforts are primarily focussed on improving the travelling experience of its customers, significantly enhancing punctuality, and providing more reliable information to the customers throughout their travels. DB aims to bring more traffic to its environmentally friendly rail network, particularly its freight transport.

Digital transformation and new technologies are changing DB’s core business. The company uses digital technologies and methods to offer attractive new products and strengthen those that it already has. Whether on the train, at a station, or on the railway, digital functioning enables it to enhance or simplify its services. In doing so, it increases its capacity and remains environmentally friendly. A 20% increase in the capacity of its rail networks has been achieved through the use of a standardised digital system. DB’s aim here is to achieve improved performance, better service quality, greater efficiency, and more growth on the rail network. A part of DB’s corporate strategy, the ‘digital railway’ also promotes the reputation of Germany as an industrially developed country.

DB Intrapreneurs is open to all employees (intrapreneurship track) and business units (called ‘co-creation’) from all parts of the organisation, from maintenance and engineering to sales. This means that employees can either apply to participate in teams and independently of their own business unit to solve validation problems and create new or improved products and services. Work in teams is always required. Operating independently of their own business unit means that teams pursue their intrapreneurial endeavours in addition to their regular jobs—with the exception of 4 workshop days which they attend within their working hours.

In both cases, DB Intrapreneurs has developed, tested, and iterated a clearly structured innovation process across four stages that see employees first become intrapreneurs and then entrepreneurs:

1. Engagement Phase: Prior to joining a batch, participants can attend several workshop and community events to generate ideas and prepare themselves before joining a batch of teams. A batch includes several teams entering the design phase together to test their problems and solutions. Every employee (and, in some cases, everyone) can participate in these events or get feedback about their ideas. The goal of this pre-batch phase is to encourage potential intrapreneurs and generate new ideas as well as lower the entrance barriers and enable a soft entry into the programme.
2. Design Phase: Across three workshops, intrapreneurs identify and validate a problem as well as develop an initial concept of a solution. During this phase, the participants must pass several gates and, if necessary, restructure and change their team to proceed. The highlight of the design phase is the Pitch Day at the end, where teams pitch before entering the build phase, which is the section where they receive funding and intensive coaching. During this phase, each team is supported by a dedicated method coach.
3. Build Phase: Over the course of three or four months, intrapreneurship teams assess how their products will behave on the market. This includes user research, service design, requirement engineering, development of first low-fidelity prototypes, business case modelling, and the drafting of a go-to-market strategy.
4. Grow Phase: If teams are able to achieve a proof of concept at the end of the build phase, they can develop their own corporate start-up. This encompasses everything, from ramping up of the organisational structures to developing and selling goods, although the process is highly unique and features the evolution of the team outside of the programme.

Workshops during the engage and design phases mostly take place in Frankfurt (Main), with some located in Berlin. Both the build and the grow phases take place in Berlin, in the Digital Base of DB. Within intrapreneurial projects, where teams of employees are allowed to work on their own ideas, such groups are supported by a venture architect. The intrapreneurial team members act as facilitators, project managers, and challengers, giving the group an overall direction. They encourage employees to set up their own corporate start-ups. Coaching includes design thinking, lean start-up, scrum, value proposition design, business modelling, and product management models.

In co-creation projects, where participants co-create together with a business unit, their role transforms. Instead of coaching employees, the members themselves serve as the co-project leads of the ventures and therefore accept partial responsibility for the success or failure of their ideas. Responsibilities are shared with the project lead of the business unit(s). The major asset of the intrapreneurial programme lies in its ability to cultivate specific capabilities of the employees and grant access to both intra- and extra-organisational networks.

There are four different exit options for intrapreneurial ventures:

1. Scaling-up of the corporate start-up inside a newly established business unit
2. Founding of a new subsidiary company wholly owned by DB where the intrapreneurs get chief experience officer positions (e.g. Chief Executive Officer or Chief Operations Officer)
3. Incorporating their business within a given business unit
4. Founding of a new start-up by the intrapreneurs (upon which they leave DB).

There is also another exit—the positive failure. The value of failing is promoted early in the innovation process. For example, if teams find out that there is no problem–solution fit, it is still a valuable and positive experience and a valuable learning tool for both the employees and DB itself. Intrapreneurs learn a large amount in a very short time, which is unprecedented among corporate training opportunities.

A number of teams have been coached and worked on a large variety of ideas. When it comes to idea generation, DB Intrapreneurs encourages participants to think globally. DB believes that ideas should be globally scalable. Thus, successful teams continue to work hand-in-hand with all business units across several silos, since DB considers that interdisciplinarity and co-creation are keys to successful innovation taking place within a corporation.

Like so many other units and companies, DB’s business units are facing digital transformation. DB Intrapreneurs believes in using and empowering the innovation potential of its employees to create the digital future of DB. Therefore, DB Intrapreneurs strives to achieve three important goals:

1. Inspire employees and business units to drive innovation by understanding digital transformation.
2. Equip employees with entrepreneurial competencies and skills to foster an innovative and entrepreneurial mindset among them.
3. Support employees as a business unit to validate and build corporate start-ups.

*The following case has been prepared in cooperation with DB. We would like to thank Florian Messner*-*Schmitt, Head of DB Intrapreneurs, and his team for their useful insights.*

### Case study 2: Obtaining digital talent through acquisition

Many companies, knowing that their current culture can make hiring or developing digital talent that they need difficult, have switched their talent acquisition strategy to buying digitally competent companies, not so much for their operations but rather for their talent.

When one of us was an angel capitalist, we made a disappointing investment wherein the entrepreneur we had invested in had a great engineering team and a good idea that was just too big for the funds and the time that were allocated for it. This entrepreneur was destined to fail. When we invested, we imagined that we could get him to begin earning revenue with a lesser product that moved in the direction of the grand dream before his funds ran out.

Unfortunately, the CEO was unwilling to work on anything other than the full version of his original dream with all its features. Once we learned that he would never change his plan, we wrote the investment off as a failure. However, we then received an offer to sell this company; this gave us a twofold return on our total investment in the firm. The buyer had no interest in the CEO’s vision or the CEO himself: the purchasing company was just buying his engineering team. Acquisition is one way to get the talent you need, and considering the team and the company that bought out the firm, I suspect it worked out well for them.

Nevertheless, simply acquiring the digital talent you need is not sufficient since you also have to keep it. In another example, one of us was running a small internet security company with a strong intrapreneurial culture and superstar engineers. To give an example of what it took to keep such talent, consider the following scenario. One of my engineers insisted on this arrangement: even though I was his boss, I could only talk to him when he arrived in the morning or left at night; under no circumstances was I allowed to interrupt his thinking between those two times. Anything I had to say to him could wait until the end of the day. He did not need or want to be managed. Once he agreed to take on a project—which was a matter of persuasion rather than command—he would take it from there.

Subsequently, we were acquired by a publicly traded company at a price of several million dollars per employee. One of the reasons we received such a high price per engineer (and a ridiculous multiplication of revenue) was that, within a week, my non-communicative engineer solved a problem the acquiring company had been working on for six months without any results. Our superstar engineer delivered a working code that got the firm’s algorithm to operate to a critical In ternet security standard. Getting engineers with that level of talent can be very valuable, and we were lucky to have had several of them.

As mentioned above, acquiring talent is not enough; you must then keep it. The acquiring company had a very different management style from ours. Their command-and-control style assumed that top management knew what was best. Two years later, none of our former employees were still working for the company that acquired us.

I heard about some of what happened when they tried their hierarchical management style on our self-motivated talent. It was difficult to get my former employees to stay long enough to cash in their stock options.

Not knowing how to nurture and support talented digital intrapreneurs makes the strategy of acquiring them useless. The same principle applies to home-grown talent. Jobs of routine processing are gradually disappearing, either becoming taken over by smart machines or getting shipped to low-wage countries. Increasingly, the jobs that remain require creativity and care—things at which people are still better than machines.

Creativity and care must come from the inside. You cannot force someone to care for their customers, since the motivation to care about them must come from the inside. As Daniel Pink points out, the same applies to creativity (TED 2009). Even rewards reduce creativity by shifting the mind from what psychologists call intrinsic forms of motivation to the extrinsic ones. The emerging kinds of work in the twenty-first century is similar to intrapreneuring and is thus in need of managers who behave more like sponsors than conventional supervisors.

This is particularly true of digital employees. Coders, for example, must make instantaneous decisions on how to structure their code and what path to take to achieve the desired result. To do that well, they must focus entirely on their intrinsic motivation and enter a state of flow. They need to be motivated by their own values instead of worrying about what their boss might think. That is why my superstar engineer asked me not to talk to him during the day. He wanted to be motivated by caring about what he was doing, by his own sense of what was right and elegant, and not by the opinion of his boss who did not really understand his code. That is a lesson for anyone who must manage digital talent. If you have hired the right people, they know more about what they are doing than you do. If you respect that, they might stay.

### Case study 3: The School for Intrapreneurs™

This case is about an online action-learning programme at a global company, which, in one year, produced a ten-to-one return and provided a proof of concept that digital intrapreneurship could yield rapid profitable results. Quick wins and the proof of a digital intrapreneurship concept is an important early step in building a culture suitable for digital intrapreneurs. Our client’s goals for the programme were as follows:

1. To increase profitable innovation in the IT sector
2. To bring out and implement bottom-up ideas
3. To develop business acumen in IT
4. To build teamwork skills in IT.

The digital intrapreneurship programme was entirely online. The design brief stipulated that no person in the programme could be required to meet with any other participant in person, which was good, since the intrapreneurial teams formed in the programme were often intercontinental, with, for example, one member based in Brazil, another one in the USA, another one in Germany, and another one in Singapore. The School for Intrapreneurs™ included four major parts:

1. *The Doorway to Intrapreneuring,* a three-hour online course covering the basics of intrapreneurship. All of the 1100 IT professionals of the company, including the head of IT, were required to complete it.
  - For managers, the course showed how to recognise and manage intrapreneurs, with case studies illustrating the role of managers as sponsors.
  - For intrapreneurs, the course inspired participants to bring out their intrapreneurial spirit and declare their desire to implement their ideas. It taught them more effective ways to move their ideas forward within a bureaucratic organisation.
  - For the company, the course located potential intrapreneurs, so that management could support the development of their ideas.
  The Doorway was run entirely by software. The company placed the software on a server and gave the participants a login. From that point on, the workshop ran without any faculty involvement. Still, the course had a 95% approval rating from graduates, which is unusually high for a required course. This illustrates the power of software and digital innovation to reduce the marginal cost of training an additional participant to almost nothing. It also illustrates more generally how digital innovation can greatly reduce operating costs.
2. *The Idea Expo* was an online forum where participants could post their ideas and get feedback from the other participants and managers. It served as an online meeting ground for forming teams around some of the ideas.
  The next step for intrapreneurs after the Expo was to move their ideas forward and attend an accelerator that would help them build a business plan for their ideas, teach them about being an intrapreneur, and build high-performance teams. At the end, it gave them an opportunity to present their ideas to senior management.
  To get into the accelerator, the participants had to form teams of three or more members, who would all be committed to the same idea. This was done to encourage team leaders to assemble their groups and form ideas that were good enough to attract at least two more members. Twelve teams progressed to the accelerator.
3. *The Pathway to Intrapreneuring* was a quick six-week online accelerator for the innovation projects coming out of the Idea Expo. Each week, there were brief lectures and readings on an aspect of intrapreneuring and building a business plan. The teams received weekly assignments and were required to write reports about how their group would address certain strategic issues.
  The assignment types included elevator pitches, building and testing rapid prototypes, managing the organisational immune system, designing and testing a business model, checking up on teamwork, developing marketing and sales plans, fostering the intrapreneurial spirit, making financial projections, and so on. At the end of each week, the teams presented their work online to two other teams, who then gave them feedback using structured forms. At the end of the accelerator, teams presented their results to a panel of executives. Six teams were funded to continue working on their innovations.
4. *The Journey to Intrapreneuring* was a twelve-week implementation workshop for the teams that were funded to develop their ideas.

As mentioned above, within the first year after the participants graduated from the *Journey to Intrapreneuring*, the programme had already produced a ten-to-one return on all the resources invested in it. Because of word of mouth, thirty more teams applied for the next round of the accelerator.

What was learned from this experiment?

1. There is a vast reservoir of creative talent and intrapreneurial spirit buried in IT departments.
2. If you demonstrate that there is a safe pathway to bring one’s ideas to management and get support for them, many digital intrapreneurs will appear. There are far potential digital intrapreneurs buried in most organisations than their management suspects.
3. The means for releasing digital innovations can itself be a digital innovation. The first two courses were delivered almost entirely as pieces of software running on a server.
4. Training intrapreneurial employees who had already been developing their innovative ideas in their own time, rather than starting with generating ideas, produced much faster and better results. This was achieved by selecting teams that had already chosen their ideas. There are generally more than enough good ideas distributed among the employee population at all times.
5. Implementation, and not idea generation, is the rate-limiting step in the innovation process. Many successful ideas had been around for quite some time but had previously lacked a pathway to implementation.
6. A process with several short cycles of rapid prototyping and business model testing and a weekly cycle of presentations caused the plans to evolve rapidly and produced better results than could have been achieved through a series of functional tests that only put it all together at the very end.
7. Implementation support after management had funded the projects was seen as quite helpful.
8. Future versions of this programme should involve more training for the management sponsors of intrapreneurial projects, perhaps as a feature of an existing high-potential leadership development programme.

## Conclusion and Implications

Intrapreneurship remains an important way to capture the creativity, excitement, and energy of entrepreneurship within a larger firm. It can let employees pursue their ideas with more resources and less personal risk than they would have if they had gone out on their own. For companies, resilient responses to a rapidly changing world require the input of a large number of intrapreneurs. The digital transformation of our society is creating challenges for existing firms and many opportunities for both entrepreneurs and intrapreneurs.

As the COVID-19 pandemic has shown, the world does not always progress smoothly. Occasionally, we face startling discontinuities. These sudden changes favour resilient firms. A firm’s capacity for responding to big changes resiliently resides in the intrapreneurs who are empowered to make all the innovations necessary for the company to adapt and create a culture to support them. However, this capacity cannot be developed overnight. It requires changing managerial attitudes and building employee trust in the fact that passionately standing up for an idea is not career-threatening (Hughes et al. 2018; Mustafa et al. 2018). Fortunately, even though building that intrapreneurial muscle is very helpful in the times of sudden change, it is also profitable in the more regular periods of the twenty-first century where rapid changes and disruption, per Moore’s law, are normal. Preparation for what Nassim Taleb calls ‘black swans’, like the coronavirus outbreak, requires many of the same steps and cultural attributes that are necessary for giving a financially informed and beneficial response to these disruptive times.

The contemporary digital world requires the development of habits of intrapreneurial innovation. It requires complete managerial acceptance of the fact that digital transformation is inevitable and that one has the choice of either being the disruptor or being disrupted.

This is not a time to cut back on innovative capacity, but rather, it is a time to expand it so that organisations can thrive in a rapidly changing world. This can be done to generate extra profits in the short term and develop the appropriate organisational systems and culture changes to face the unknown shocks that the future will surely bring.

The benefits of digital intrapreneurship are not just in the new products, but also in the better ways for delivering existing goods and services, often with fast results. Digital intrapreneurship creates opportunities for more intrapreneurs than the traditional applications of intrapreneurship. There are many more high-ROI opportunities to improve the way things are done with digital technology today than there were opportunities to develop new products and services in the industrial era.

This chapter has identified several ways in which companies can surface, choose, and empower digital intrapreneurs. It has shown how the exploitation of new business opportunities can be speeded up. It has also identified more effective ways of operating digitally in the non-digital business areas. Moreover, it has displayed that digital intrapreneurship is needed to reduce the risk of being disrupted by entrepreneurial competition.

We have shown several ways in which companies can bring out and support potential digital intrapreneurs. We have provided the means for distinguishing true digital intrapreneurs who can be trusted from the ‘promoters’ who are talented speakers that lack the character to persistently work hard and persevere through the difficult times until the eventual implementation of their ideas.

Creating systems and a corporate culture for supporting digital intrapreneurs is a core competency for the times of rapid digital transformation. Some ways of doing that include a clear organisational vision for digitalisation, valuing, training, and supporting intrapreneurs, more managers serving as effective sponsors, empowered cross-functional teams, high risk tolerance, failure analysis, increased cross-organisational generosity, acceptance of small beginnings, discretionary resources allocated to lower levels, and less reliance on command-and-control managerial styles and more on inspiration, coaching, and vision.

## Footnotes

1 For exceptions, see Covin et al. (2020), Hughes et al. (2018), Kraus et al. (2019), Marvel et al. (2007), Monsen et al. (2010), Mustafa et al. (2018).
